# Influencing Factors of Negative Motivation in College Students' English Learning Relying on the Artificial Neural Network Algorithm

**DOI:** 10.1155/2022/2323870

**Published:** 2022-10-17

**Authors:** Ping Liu

**Affiliations:** Foreign Language School, Hubei Polytechnic University, Hubei, China

## Abstract

College English has received increasing focus as an important part of the education system. However, the continuous development of English instruction has not simultaneously promoted students' positive learning motivation for English courses. The generation and growth of negative motivation have become a common problem among college students. Students' enthusiasm for learning English courses is gradually fading and teachers' teaching value has also become difficult to guarantee, which seriously affects the normal and orderly progress of education and teaching activities. Therefore, it is very important for the healthy development of English teaching to understand and study the affecting elements of negative motivation in English learning of university students and to provide scientific and effective suggestions for teachers and learners to establish a good teaching and learning attitude. Relying on the interpretation of a negative motivation theory, this paper studies various influencing factors by means of the artificial neural network algorithm. The principal component analysis method is introduced to improve the traditional BP algorithm in terms of the frequency of iterations and the length of computation time, which realizes the accurate and efficient analysis of college students' English learning data. The results of the analysis revealed that the comprehensive error of this algorithm in the analysis of influencing factors was in the range of 0.004 to 0.012. Through the calculation of the eigenvalues and cumulative contribution rate of negative motivation influencing factors, it is found that factors such as the curriculum setting, teaching method, and teacher-student relationship have the greatest influence on students' negative motivation in English learning. The eigenvalues were 1.027, 1.319, and 1.422, respectively. The cumulative contribution rate reached 64.57%, 26.11%, and 23.62%, respectively. From this aspect, it is necessary to improve these aspects in order to eliminate the negative motivation of learning.

## 1. Introduction

With the development of college English instruction, the status and influence of learning motivation in college students' English learning activities are becoming more and more prominent. As a major factor in guiding college students' English learning behavior, learning motives include not only positive motives but also negative motives. In the current stage of English education, the negative motivation of students' English learning has been significantly enhanced and has gradually become a common phenomenon. More and more students are unable to face English learning positively. This is not conducive not only to the sound growth of English instruction but also to the cultivation of foreign language talents with high comprehensive quality. Therefore, it is very important to study the affecting elements of college students' negative motivation in English learning and understand the generation of negative motivation to enhance students' positive motivation for learning and promote the sustainable development of English teaching. The artificial neural network (ANN) algorithms have received a great deal of attention and exploration over the past few years and have been successfully applied in a wide range of industrial fields, giving ample play to their own unique strengths and application value. For example, it can be seen in medical diagnosis, machinery manufacturing, construction engineering, finance, insurance, and other industries. By relying on artificial neural network intelligent algorithms, various industries have also ushered in unprecedented development opportunities in the market and achieved considerable development results. In the study of the affecting elements of negative motivation in English learning among university students, it can comprehensively judge and effectively analyze the entire English learning process of students and draw accurate conclusions based on scientific basis, which has a very important practical value to the improvement and promotion of college English instruction.

Regarding the affecting elements of negative motivation in English learning among university students, numerous academics have carried out in-depth studies. Batubara et al. believed that the learning environment is very important for the cultivation of students. They used the Montessori method to explore the factors that affect students' negative motivation in learning [1]. Raczoski et al. used the expectation-value-cost motivation model to study the motivation and influencing factors of students studying abroad courses. They stated that external effort cost and self-efficacy are negative motivation factors [2]. Hasan investigated the changes in learners' motivation to study English as a second language under the background of Indonesian universities and found out the factors that affect the changes in their negative motivation [3]. Anjum et al. showed that there is a negative and low correlation between the influence of the motivation level and students' English academic performance through the Pearson coefficient correlation survey [4]. Soriano et al. selected participants from various programs at Quirino State University, Cabalogis Campus in the Philippines and explored the students' life experiences and the factors that affect their negative motivation to learn English [5]. Yarlagadda et al. used the Bayesian network analysis method to explore the key factors affecting the negative motivation of college students' English learning. They said that the responsibilities of a learner and the properties of a learner had the greatest adverse effect on it [6]. These studies have used different methods to study the influencing factors of negative motivation in English learning, but the conclusions are not inductive and fail to reveal the complexity of each influencing factor. The research studies on the influencing factors of negative motivation require more accurate analysis results from colleges and universities, for which the artificial neural network algorithm is a good choice.

As a major branch of the intelligent algorithm, the artificial neural network algorithm has a very important application value. Ge investigated the affecting elements of basketball damage in sports instruction based on the ANN and analyzed the causes of knee damage in basketball training in general universities [7]. Allahyari E used an artificial neural network model to analyze the influence of different factors, such as gender, age, education level, and place of residence on people's emotional intelligence level [8]. Dar investigated local antitumor effects using ANN modeling and developed a neural network time series model to evaluate it [9]. Palanichamy used artificial neural network algorithms to detect motor failures. Compared with traditional methods, it can not only increase the new entry detection function but can also improve the accuracy and stability of system performance [10]. Golnaraghi et al. analyzed the significant impact of changes in labor productivity on the economy through artificial neural networks and then proved through experiments that this method is more useful than statistical regression techniques [11]. Hu et al. built a postmodern media communication perspective system by establishing an in-depth research and learning platform for college marketing based on the AI neural network [12]. On the whole, the ANN algorithm has been widely used in the market, but there are few studies that combine it with the elements that impact negative motivation in English studying among university students. Therefore, it is very necessary to use the ANN algorithm to analyze the affecting elements of college students' negative motivation in English learning, which is important to promote the healthy growth of English education.

This paper relies on the ANN algorithm to study various influencing factors of negative motivation. Through research and investigation, it was found that among 146 students, a total of 113 students had negative motivation in English learning, accounting for about 91.1% of the total number. When analyzing the influencing factors of negative motivation, the error range between the analysis value of the traditional BP algorithm and the actual value was 0.016 to 0.275, while the error range of the PCA-BP algorithm improved by principal component analysis was 0.004 to 0.008 in this paper. It showed that the performance of the improved algorithm in this paper is more rational in the analysis of influencing factors. The improved algorithm was used to calculate the eigenvalue and cumulative contribution rate of the influencing factors, and it was found that the eigenvalue of the curriculum setting factor reached 1.027. The cumulative contribution rate was 64.57%, which had the most significant impact on negative learning motivation.

## 2. Influencing Factors of Negative Motivation in English Learning

### 2.1. Overview of Negative Motivation in English Learning

Negative motivation is an important content in the study of learning motivation. There is a close relationship between learning motivation and negative motivation; so, it is necessary to understand learning motivation before studying the influencing factors of college students' negative motivation in English learning [13].

**Figure 1:**
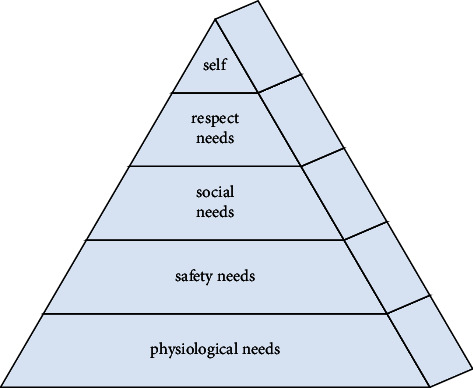
The hierarchy of needs.

The study of learning motivation originally emerged in the field of psychological studies in the 1930s and has since progressively extended into the area of pedagogical studies. Although the study of learning motivation is becoming a century old, a sufficiently precise and widely accepted definition is yet to be developed owing to the inherent intricacies of learning motivation and the diverse areas of study, perspectives, and standpoints of various academics. Scholars have given different definitions of motivation from different perspectives, which to a certain extent allow people to understand the nature of learning motivation more clearly and comprehensively. In terms of the hierarchy of needs theory, motivation for learning is intrinsic to people's study actions. It is characterized by internal elements and is also influenced by external elements, as shown in Figure 1. Under the combined effect of internal and external elements, study motivation not only can boost, enhance, sustain, and adjust an individual's studying actions but can also reduce an individual's original degree of study motivation due to the influence of some negative factors. Because of the influence of negative factors, the phenomenon that students' original learning motivation level declines is called the generation of negative learning motivation.

The generation of negative learning motivation can be viewed as a passive aspect of motivation, which is a passive manifestation expressed in the learner's study behavior. From a certain point of view, the generation of negative learning motivation can be regarded as a slow dynamic process, that is, the process of students' English learning positive motivation is gradually weakening. There is an inseparable relationship between the weakening or elimination of college students' motivation in the process of English learning and negative motivation. Analyzing the influencing elements of negative motivation is the most important means and method for teachers to improve their own work and students to reflect on their learning effects. For example, teachers should think about which links in teaching activities will affect students' enthusiasm for learning English; students reflect on their own learning methods and states in the learning process.

The factors that affect the occurrence of negative motivation for learning can also be divided into internal reasons and external reasons. External reasons refer to various factors that will negatively affect students, which exert a negative effect on learners' learning behavior through internal factors, with a significant impact on negative motivation, while internal factors are the main reasons for the decrease in learners' motivation, including self-confidence, attitude, and personality traits. It can be seen from numerous studies that those with strong self-confidence in learning and positive attitudes are slightly less affected by external elements, while people with insufficient self-confidence or negative attitudes are more likely to be influenced by adverse external elements. Therefore, it is believed that of the two elements that influence a decline in motivation, the internal elements of the learner are supposed to be the main cause, while the external influencing elements use the internal elements to affect the motivation of the learners and the size of the influence varies from person to person.

### 2.2. The Artificial Neural Network Algorithm

**Figure 2:**
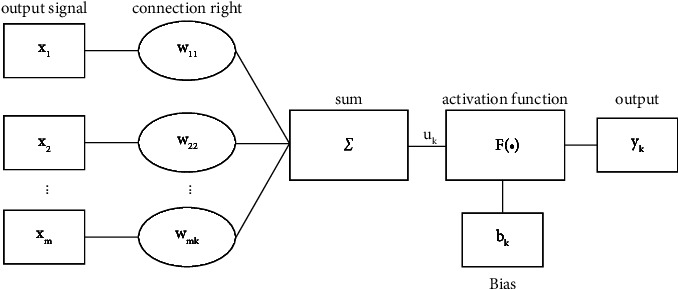
Artificial neuron model.

In order to better understand the impact of these internal and external elements on learning negative motivation, this paper combines the ANN algorithm to conduct research studies. As a method for simulating the biological neural network, the ANN algorithm is similar to the human nervous system, the main component of which is neurons, as shown in Figure 2. These neurons have a very important role, which are like nerve cells in the human nervous system. Neural modules, such as dendrites, axons, and synapses, with different functions are composed of many nerve cells; each of which performs its own function and cooperates with each other. In the analysis of the influencing elements of college students' English learning negative motivation, even if there are factors with high complexity, their function can be realized.

The research on the influencing elements of learning negative motivation is not a simple linear process relying on algorithms but a complex and intersecting processing method. The BP network is a highly nonlinear network, which generally refers to those multilayer forward neural networks that use the error back propagation algorithm, that is, the BP algorithm, and is currently the most widely used neural network in scene problem analyses [14].

In this method, the acquisition, analysis, and identification of the influencing factors of learning negative motivation information are carried out by its algorithm.

**Figure 3:**
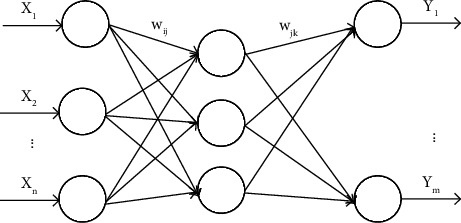
BP network topology.

Its main components are input layer, hidden layer, and output layer. In addition, it is necessary to use the BP algorithm to continuously adapt and train the weights and thresholds. The selection of the weights and thresholds of the BP network structure has been repeatedly adjusted and trained by the BP algorithm. The topology of the BP network is shown in Figure 3:

In Figure 3, the input value of the student's English learning data is *X*_1_, *X*_1_, ⋯*X*_*n*_ and the output value is *Y*_1_, *Y*_1_, ⋯*Y*_*m*_. The interpretation of each parameter is shown in Table 1.

The BP network function mapping relationship is mainly the mapping from an independent variable to a dependent variable. The specific training steps are divided into 6 steps.

First, network initialization is performed and the threshold sum of the network is initialized.

Second, the output value of the hidden layer is calculated. It is supposed that the output variable is *X*, and the thresholds and weights in the network input and hidden layers are *a* and *w*_*ij*_, respectively, which can be expressed as formula (1) [15].

(1) $$\begin{array}{c} H_{j}=f\left(\overset{n}{\underset{i=1}{\sum }}w_{ij}x_{i}-a_{i}\right),j=1,2,\cdots ,l\text{.} \end{array}$$

In formula (1), *l* is the number of hidden layer nodes. *f* is the activation function of the hidden layer.

Third, the output layer is calculated. *H* represents the output of the hidden layer. *w*_*jk*_ and *b* represent the connection weight and threshold of the network, respectively. The formula for predicting the output of negative motivation factors is formula (2) [16]:

(2) $$\begin{array}{c} H_{k}=\overset{l}{\underset{j=1}{\sum }}H_{j}w_{jk}-b_{k},k=1,2,\cdots ,m\text{.} \end{array}$$

Fourth, the output error is calculated with reference to the student learning data. Its calculation is mainly carried out through the expected output and predicted output of the negative motivation factors. These two terms are denoted as *Y* and *H*, respectively. The error calculation formula is expressed as formula (3) [17].

(3) $$\begin{array}{c} e_{k}=Y_{k}-H_{k},k=1,2,\cdots ,m\text{.} \end{array}$$

Fifth, the network input layer and hidden layer weights are updated. The network connection weights *w*_*ij*_ and *w*_*jk*_ are updated with the prediction error of the network *e*, as shown in formula (4):

(4) $$\begin{array}{c} w_{ij}=w_{ij}+\eta H_{j}\left(1-H_{j}\right)x\left(i\right)\overset{m}{\underset{k=1}{\sum }}w_{jk}e_{k},i=1,2,\cdots ,n\;j=1,2,\cdots ,m\text{.} \end{array}$$

In formula (4), *η* is the learning rate.

The sixth step is that the algorithm iteratively judges whether the factors affecting the generation of negative motivation in the English learning data predict the complete end. If it is not over, it will go back to Step 2 until it is over.

However, in this process, the shortcomings of the BP neural network are also very obvious. The first is that the convergence process of the BP neural network model is slow, which leads to the long iterations and computing time required for the training of negative learning motivation factors. The number of layers is difficult to determine. In particular, the number of layers of neurons in the hidden layer of the model and the number of neurons in each layer can only refer to the settings of the empirical structure. This method often causes a lot of computational redundancy in the training of the BP neural network, which affects the normal analysis of learning data.

**Figure 4:**
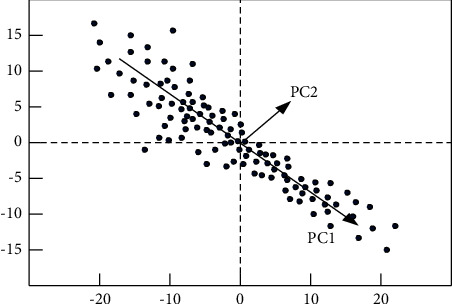
Principle of the PCA method.

These problems limit the analysis and research of the BP neural network model on the factors affecting students' negative motivation in English learning to varying degrees. Therefore, this paper uses the PCA method to improve it. PCA is a global algorithm, also known as the principal component analysis method. Its main advantage is that it can provide more information even in the context of less data. It is mainly a basic method to simplify multivariate by linear combination of original variables, as shown in Figure 4.

*X* is supposed to be the observed student learning data matrix. Each row of it represents an observation and each column represents a variable indicator. These variable indicators will affect the intensity of students' negative motivation to learn at different levels. Among them, there are *X*_1_, *X*_2_, ⋯,*X*_*p*_ variables, with a total of *P*, and the linear combination of these variables is shown in formula (5) [18].

(5) $$\begin{array}{c} \left\{\begin{array}{c} Y_{1}=a_{11}X_{1}+a_{12}X_{2}+\cdots +a_{1p}X_{p}\\ Y_{2}=a_{21}X_{1}+a_{22}X_{2}+\cdots +a_{2p}X_{p}\\ \begin{array}{c} \cdots \cdots \cdots \\ Y_{p}=a_{p1}X_{1}+a_{p2}X_{2}+\cdots +a_{pp}X_{p} \end{array} \end{array}\right.\text{.} \end{array}$$

Among them, if *P* comprehensive indexes *Y*_1_, *Y*_2_, ⋯,*Y*_*p*_ are obtained, the matrix transformation needs to meet the conditions [19].

Unit vectors are all coefficient vectors, as shown in formula (6) [20].

(6) $$\begin{array}{c} a_{i}^{\prime }=\left(a_{i1},a_{i2},\cdots ,a_{ip}\right)\text{,} \end{array}$$

*a*_*i*_′*a*_*i*_=1, which satisfies formula (7):

(7) $$\begin{array}{c} a_{i1}^{2}+a_{i2}^{2}+\cdots a_{ip}^{2},\left(i=1,2,p\right)\text{.} \end{array}$$

There is no relationship between *Y*_*i*_ and *Y*_*j*_(*i* ≠ *j*, *i*, *j*=1,2, ⋯, *p*).

*Y*_1_ is the variance max in all linear combinations of *X*_1_, *X*_2_, ⋯, *X*_*p*_. *Y*_2_ is the variance max in all linear combinations of *X*_1_, *X*_2_, ⋯, *X*_*p*_ uncorrelated with *Y*_1_. *Y*_*p*_ is the variance max in all linear combinations of *X*_1_, *X*_2_, ⋯, *X*_*p*_ uncorrelated with *Y*_1_, *Y*_2_, ⋯, *Y*_*p*−1_.

From these conditions, the first main ingredient, the second main ingredient ⋯the *m*-th main ingredient in the influencing factor category are obtained in turn.

If the first *m* principal component is selected to replace the original *p* independent variables (*m* < *p*), the dimension of the data will be reduced.

The steps are divided into the following 4 steps:

The first step is to calculate the original data covariance matrix ∑. The solution of the eigenvalues of matrix ∑ is as formula (8):

(8) $$\begin{array}{c} \lambda_{1}\geq \lambda_{1}\geq \cdots \geq \lambda_{p}>0\text{.} \end{array}$$

The corresponding unit eigenvector is *a*_1_, *a*_2_, ⋯, *a*_*p*_. The variance of the *i*-th principal component is *λ*_*i*_, and *i*=1,2, ⋯, *p*.

The next step is to calculate the variance contribution of each main ingredient, as shown in formula (9):

(9) $$\begin{array}{c} \varphi_{i}=\frac{\lambda_{i}}{\sum_{j=1}^{p}\lambda_{j}},i=1,2,\cdots ,p\text{.} \end{array}$$

The variance contribution rate of each main ingredient reflects the amount of information. The reflection of the information content of the principal components is weakened in turn. At the same time, the cumulative variance contribution rate of the variance of the first *M* main ingredients is calculated as formula (10):

(10) $$\begin{array}{c} \Psi_{m}=\frac{\sum_{i=1}^{m}\lambda_{j}}{\sum_{j=1}^{p}\lambda_{j}},m<p\text{.} \end{array}$$

Among them, when the cumulative variance contribution rate reaches more than 20%, it is considered that this factor has a greater impact on the negative motivation of English learning. If the value reaches more than 60%, it indicates that the influencing factors have a significant influence on negative motivation. At this time, the original *p* variables can be replaced by *m* principal components.

Finally, the scores of the observed samples on the principal components are calculated as formula (11):

(11) $$\begin{array}{c} Y_{i}=a_{11}X_{1}+a_{12}X_{2}+\cdots +a_{1p}X_{p},i=1,2,\cdots ,m\text{.} \end{array}$$

If these four steps are satisfied, the principal component analysis can be performed by converting the original dimensional data into the dimension-reduced dimensional data. Similarly, the higher the score is, the more prominent the influence of such factors in the negative motivation of English learning will be.

### 2.3. Evidence of Influencing Factors

For the purpose of testing the effectiveness of the research on the affecting elements of college students' negative motivation in English learning relying on the ANN algorithm, this paper investigated the negative motivation of 2021 students in a university. Then, the PCA-BP neural network algorithm was used to analyze the influencing factors of negative motivation on the survey sample data. It was compared with the traditional BP neural network algorithm to verify the feasibility of this method.

Questionnaire survey: This paper conducted a questionnaire survey on the students of the four majors of tourism management, primary education, civil engineering, and computer science in the 2021 grade of the university. The college English course of the school belonged to the compulsory courses of these 4 majors. Students needed to complete 6 credits in 1 academic year to meet the English study requirements. For the purpose of guaranteeing the usefulness of the study, this paper randomly selected 158 students from these four majors as the object of investigation on the influencing factors of negative motivation in English learning. The questionnaire data and details of the experimental subjects are shown in Tables 2 and 3.

It can be seen from Table 2 and Table 3 that the reliability coefficient of the questionnaire in this paper reached 0.862, which met the reliability standard (Generally speaking, if the reliability coefficient of the questionnaire is below 0.6, it means that the reliability of the questionnaire needs to be verified. If the reliability coefficient is above 0.8, it means that the reliability of the questionnaire is good). The total number of boys selected in the experiment was 78 and the total number of girls was 68. The ratio of male to female was also relatively balanced.

**Figure 5:**
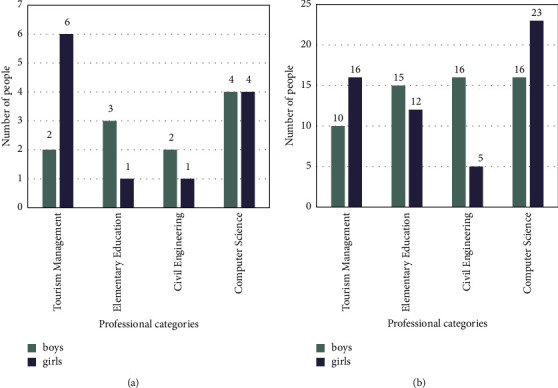
A survey of negative motivation in English learning.

The survey of English learning negative motivation of students of various majors is shown in Figure 5.

Figure (a)5 shows the number of people who did not generate negative motivation. Figure (b)5 shows the number of people with negative motivation.

It can be seen from Figure 5 that among the subjects of each major, the number of people who have negative motivation for English learning was far more than the number of people who did not have negative motivation. According to the questionnaire data, among the 146 students, a total of 113 students had negative motivation in English learning, accounting for about 91.1% of the total number. Among them, there were 26 people majoring in tourism management, accounting for 76.5% of the total number. There were 27 students majoring in primary education, accounting for 79.4% of the total. There were 21 people majoring in civil engineering, accounting for 91.3% of the total number. There were 39 people majoring in computer science, accounting for 68.4% of the total number. In general, most students were seriously affected by negative motivation in English learning, which hindered the growth of learning enthusiasm and disrupted the orderliness of learning activities. This kind of performance was more obvious in the civil engineering profession, which might be related to the professional characteristics. The gender ratio of this major was heavily biased towards boys, so the trend of negative motivation in English learning was more obvious, which also proved the necessity of the experiment in this paper.

**Figure 6:**
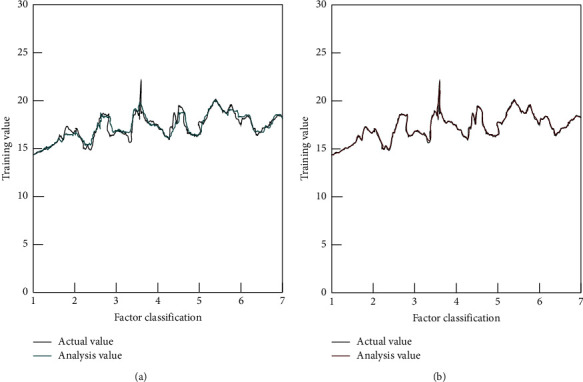
Analysis of influencing factors of negative motivation.

Data analysis: The survey data of these 113 students with negative motivation to learn were used as an independent sample for the algorithm analysis. The BP algorithm and the modified PCA-BP algorithm in this paper were used to analyze and study the influencing factors of negative motivation. In the training process of the two algorithms, the training set was used as the input of the neural network. The target error of the network was set as goal = 0.01, and the training times were set as 10,000 times. The learning rate was set to 0.75. The sigmoid tangent function tansig was selected as the neuron function of the hidden level and the output level in the network and the training function of the network was training. The MATLAB software (2022a version) was used to establish an analysis model for the influencing factors of negative motivation in English learning, and the results are shown in Figure 6.

Figure (a)6 shows the analysis of the BP algorithm. Figure (b)6 shows the analysis of the PCA-BP algorithm.

According to the survey items, the influencing factors of students' negative motivation in English learning are divided into 7 categories, namely, teaching equipment factors, curriculum setting factors, teaching method factors, teaching material factors, teacher-student relationship factors, learning difficulties factors, and personality factors. As can be seen from Figure 6, the traditional BP neural network algorithm was not very satisfactory in the overall analysis of the negative motivation factors in the survey data. The first four items, as external factors, are of objective nature, and the complexity of the influencing factors is not high. The error range between the analytical results and the actual results of the traditional BP algorithm was 0.016 to 0.275. The error range of the modified algorithm in this paper was 0.004 to 0.008. The latter three are internal factors. In the analysis of the affecting elements of negative motivation, their correlation is wider and the complexity is higher, and they vary due to individual differences of students. The error range between the analytical value and the actual value of the traditional BP algorithm was 0.019 to 0.304. The error range of the improved algorithm in this paper was 0.008 to 0.012.

In the process of students' learning, due to the setting or changes of some external conditions, it is very likely that some students' English learning motivation will be weakened. From the survey data of this paper, if the English teaching equipment, teaching materials, curriculum, and teaching methods are not planned scientifically and systematically, it will easily lead to the weakening of students' motivation. However, internal factors such as the teacher-student relationship, learning difficulties, and students' personalities have no weaker effect on negative motivation than external factors, which are often more complex. Without an effective analysis of the relationship between these factors, it is impossible to provide scientific decision-making to improve the negative status quo of English teaching and learning. From the analysis error, it can be seen that the improved PCA-BP algorithm in this paper can analyze the influencing factors more accurately.

**Figure 7:**
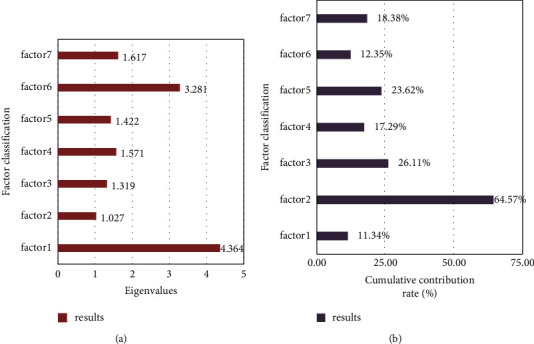
Eigenvalues and cumulative contribution rates of various factors.

In order to further understand the specific impact of these factors on negative motivation, this paper uses the PCA-BP algorithm to calculate the eigenvalues and cumulative contribution rates of various factors in the learning process, as shown in Figure 7.

Figure (a)7 shows the characteristic values of the influencing factors. Figure (b)7 shows the cumulative contribution rate of influencing factors.

Generally speaking, an eigenvalue greater than 1 indicates that the factor selection is reasonable. The greater the cumulative contribution rate is, the greater the effect of the influencing factor will be. It is evident from Figure 7 that the characteristic values of the seven types of factors were 4.364, 1.027, 1.319, 1.571, 1.422, 3.281, and 1.617, respectively, which were all greater than 1. It showed that in the experiment of this paper, the factors screened by the survey have a significant impact on negative motivation. The cumulative contribution rates are 11.34%, 64.75%, 26.11%, 17.29%, 23.62%, 12.35%, and 18.38%, respectively. Among them, the cumulative contribution rate of curriculum setting is the largest, followed by teaching methods, teacher-student relationship, student personality, learning difficulties, and teaching equipment.

According to the data analysis, the curriculum setting elements have the most significant impact on the negative motivation of students' English learning, which was much higher than that of other factors. The reason why the curriculum setting factor has become the most influential factor is mainly related to the large reduction of college English class hours in recent years and the nature of college English courses. This greatly exacerbates the contradiction between the time-consuming and labor-intensive characteristics of college English learning and the serious shortage of teaching hours. The mechanical repetition of teaching form and content will inevitably lead to students losing interest in learning, making them feel tired of learning and creating negative motivation to learn English. The two influencing factors of teaching style and teacher-student relationship also have a great influence on negative motivation. A single boring teaching method and a negative teacher-student relationship will make students' enthusiasm for English learning subside. Although students' personality, teaching methods, learning difficulties, and teaching equipment have less impact on negative motivation than the first three items, they also have important reference value in improving the English teaching and learning process.

## 3. Conclusion

Among college students, as a relatively common negative phenomenon, the occurrence of negative motivation in English learning poses a great challenge to the value and significance of English education and teaching. Combined with the ANN algorithm, this paper examines the influencing factors of students' negative motivation in learning and finds that factors such as curriculum setting, teaching methods, and teacher-student relationship have a great influence and correlation with the generation of negative motivation. If the curriculum and teaching are not organized scientifically and effectively or a good and a harmonious relationship between instructors and learners is not formed, the chances of students losing their enthusiasm and motivation for English courses are greater. Therefore, for the purpose of ameliorating these problems, it is important to combine the teaching practice from the perspective of these influencing factors to raise the instruction level and learning quality, so as to eliminate the negative motivation of students. Due to some objective reasons, there are still several areas that need to be improved in this paper, and the experimental level of this paper is also limited. Only one university was selected as the sample, and more differentiated and diversified sample data were not introduced, which made the conclusions of this paper still have certain limitations. In future research, algorithms will be combined to explore the influencing factors of negative motivation from different perspectives and scopes.

## Figures and Tables

**Table 1:** Interpretation of each parameter.

Sequence	Parameter	Paraphrase
1	*w*_*ij*_	BP network weight
2	*w*_*jk*_	BP network weight
3	*n*	Network input node
4	*m*	Network input node

**Table 2:** Details of the experimental questionnaire.

Sequence	Project	Data
1	Number of releases	158
2	Number of recycling	146
3	Efficient	92.4％
4	Number of questions	16
5	Reliability coefficient	0.862

**Table 3:** Details of the subjects of this paper.

Professional category	Number of boys	Number of girls	Total
Tourism management	12	22	34
Elementary education	18	13	31
Civil engineering	18	6	24
Computer science	30	27	57

## Data Availability

The data used to support the findings of this study are available from the corresponding author upon request.
